# Word-object and action-object association learning across early development

**DOI:** 10.1371/journal.pone.0220317

**Published:** 2019-08-08

**Authors:** Sarah F. V. Eiteljoerge, Maurits Adam, Birgit Elsner, Nivedita Mani

**Affiliations:** 1 Psychology of Language, University of Goettingen, Goettingen, Germany; 2 Leibniz ScienceCampus “Primate Cognition”, Goettingen, Germany; 3 Developmental Psychology, University of Potsdam, Potsdam, Germany; University of Maryland, UNITED STATES

## Abstract

Successful communication often involves comprehension of both spoken language and observed actions with and without objects. Even very young infants can learn associations between actions and objects as well as between words and objects. However, in daily life, children are usually confronted with both kinds of input simultaneously. Choosing the critical information to attend to in such situations might help children structure the input, and thereby, allow for successful learning. In the current study, we therefore, investigated the developmental time course of children’s and adults’ word and action learning when given the opportunity to learn both word-object and action-object associations for the same object. All participants went through a learning phase and a test phase. In the learning phase, they were presented with two novel objects which were associated with a distinct novel name (e.g., “Look, a Tanu”) and a distinct novel action (e.g., moving up and down while tilting sideways). In the test phase, participants were presented with both objects on screen in a baseline phase, then either heard one of the two labels or saw one of the two actions in a prime phase, and then saw the two objects again on screen in a recognition phase. Throughout the trial, participants’ target looking was recorded to investigate whether participants looked at the target object upon hearing its label or seeing its action, and thus, would show learning of the word-object and action-object associations. Growth curve analyses revealed that 12-month-olds showed modest learning of action-object associations, 36-month-olds learned word-object associations, and adults learned word-object and action-object associations. These results highlight how children attend to the different information types from the two modalities through which communication is addressed to them. Over time, with increased exposure to systematic word-object mappings, children attend less to action-object mappings, with the latter potentially being mediated by word-object learning even in adulthood. Thus, choosing between different kinds of input that may be more relevant in their rich environment encompassing different modalities might help learning at different points in development.

## Introduction

The fetus can hear the rhythm and intonation of voices in the immediate surrounding [1], might feel the mother move, and moves by itself [2], thereby experiencing language and movement during the latter period of gestation. As soon as the infant enters this world, she is confronted with a complex, multimodal environment encompassing both of these information types [3, 4]. This rich environment can help familiarize the child with the conceptual and social aspects of their world where successful communication encompasses both the comprehension of spoken language and observed actions. While infants seem to show similar patterns of learning for word-object and word-action mappings [5], word-object and action-object mappings have only been examined in older age groups (e.g., 30-month-olds; [6]). In the current study, we examine children’s learning of word-object and action-object associations throughout infancy and early childhood. Our aim is to compare learning from these different domains, since information from these domains are present in the input that is provided to the child from early on and at least early learning of such associations can be captured by comparably simple associative learning.

Much research has shown how early and quickly children acquire language, understanding a few words long before their first birthday around six months of age (e.g., [7–9]). At a similarly young age, infants seem to perceive actions and can remember them after seven weeks [10], although there is some debate as to the extent to which young infants are able to discriminate between matching and mismatching action-object associations (e.g., a woman using a hairbrush to brush her hair vs. her teeth at 5.5 months [10] and at 6 months [11]).

Auditory and visual information often co-occur in the child’s learning environment: For example, Gogate et al. showed that 76% of play interactions contained synchronous, temporally aligned bimodal object namings during the prelexical phase (between 5 and 9 months) with this number dropping to 59% in the early (9—17 months) and 36% in the advanced lexical (21—30 months) stage [3], with potential similarities and differences n multimodal motherese across different cultures [12]. This mirrors the close relationship of words and actions with objects and shows how omnipresent their co-occurrence is, especially during the first year of life. Against this background, we examine whether the acquisition of word-object and action-object associations proceeds at a similar time scale in early childhood?

In the following, we will review the literature to-date on the learning of word-object and action-object associations. Thus far, there has been little emphasis on examining the similarity of the trajectory of learning of these two kinds of associations in early development. The finding of similar trajectories in different domains would speak to the debate about potentially shared underlying resources driving learning in both domains. Note that, given the focus of the current study, we focus here exclusively on the learning of word-object and action-object associations, and do not include the extensive literature on the learning of word-action associations, i.e., verb learning, although we note it is possible that both word-object and action-object associations are likely to be impacted by verb learning. Note also that we predominantly refer to the literature of action-object learning and not function-object learning given that the actions in this study were not affordances or functions of the objects themselves.

The general consensus in the literature to-date appears to be that, at later ages, from around 3 to 4 years, there are few differences in children’s ability to fast-map novel word-object and action-object associations [6, 13, 14]. Riggs, Mather, Hyde, and Simpson showed that a) 3- to 4-year-old children choose a novel object relative to a familiar object when presented with a novel action or a novel word, b) retain the action-object association and extend it to other members of the object category as they do with words, and c) distinguish between a previously familiarized novel-action-object mapping and an unfamiliar novel-action-object mapping, showing that they had formed a detailed representation of the novel action presented [13]. In a more recent study, Dysart, Mather, and Riggs familiarized 3- and 4-year-olds with two novel objects. In the following test phase, the two—now familiar—objects were presented alongside another novel object (“super-novel”), accompanied by a novel label or action. In both conditions, children chose the super-novel object, suggesting that novelty drives referent selection in both word-object and action-object mapping [14].

Directly contrasting learning of word-object and action-object mappings, Childers and Tomasello (2003) taught 30-month-old children novel word-object and action-object mappings and found reliable learning of action-object mappings from early on, as well as extension of learning action mappings to other members of the object category [6]. Taken together, these studies suggest that word-object and action-object association learning overlap considerably and might, therefore, develop in synchrony.

However, other studies have shown that action-object representations may indeed be prioritized relative to word-object mappings. One reason for this, suggested by Yoon, Heinke, and Humphreys, is that action representations can be accessed merely via a visual route in contrast to labels that require additional linguistic information (i.e., semantics) for successfully mapping the representation with the object [15]. Work with adults finds that adults are faster to reach action decisions on objects (what kind of action can be performed on an object) relative to words, which is interpreted as suggesting that access to action knowledge about objects is facilitated compared to access to semantic knowledge [16]. Hahn and Gershkoff-Stowe, testing 2- and 3-year-old children and adults, found that adults and young children retrieve newly learned action-object representations easier than word-object representations [17]. Further, Dysart, Mather, and Riggs found that the presence of known competitors (e.g., hairbrush) supported the retention of action-object mappings after 7 days but not the retention of word-object mappings at 50 months of age [18].

However, in contrast to some of the action literature reported above, Puccini and Liszkowski report that, at 15 months of age, children only learned the mapping between a word and object, and not an action and an object, and only when the word was presented without an accompanying referential action [19]. This might suggest that action-object mappings may not be as robust as word-object mappings due to potentially greater variability in the former relative to the latter (i.e., a ball can roll, fly, and bounce, but is usually only called a “ball”). This finding, along with numerous studies of word-object mapping in early childhood (see [20] for a review) may be taken to suggest that early in life, children may tend to prioritize the mapping of words to objects and that auditory input dominates the scene in multimodal naming [19, 21, 22]. However, given the contrasting results from the action learning literature, the picture of learning words and actions for objects still appears to be diffuse and requires systematic investigation.

Taken together, while infants are able to recognize previously experienced associations between actions and objects and words and objects, in a multimodal environment, encompassing both of these information types, the complexity of the input might lead to children prioritizing one information type over the other in mapping information onto objects. Accordingly, children might learn either word-object- or action-object mappings at different stages in early development, and only later be able to integrate both mappings into their object knowledge. Alternatively, if children learn about both information types already from early on, this would support the idea of a similar developmental trajectory of word-object and action-object association learning in early childhood.

### Current study

Against this background, the current study examined the extent to which participants learn to associate words with objects relative to actions with objects, when given the opportunity to learn both. Thus, we presented participants with distinct action-object (Object A-Action A/Object B-Action B) and word-object mappings (Object A-Word A/Object B-Action B) for novel objects and examined differences in their learning of these at the different ages tested. In a first learning phase, participants saw two novel objects and heard distinct labels for these objects and saw distinct actions being performed on each of the objects in a temporally asynchronous manner. In a following test phase, they first saw static pictures of both objects presented simultaneously on-screen. The objects then disappeared from the screen at which point participants either heard the label in a carrier noun phrase or saw the action associated with one of the objects. Subsequently, participants saw both static pictures of the objects on-screen again and we examined their fixations to the target object as an index of their learning of the word-object and action-object association. We tested children at 12 months, 24 months, 36 months of age, as well as adults, to capture the early developmental trajectory of word-object and action-object learning.

Based on the literature on the salience of word-object associations in early childhood (e.g., [23]), we predicted that 12-month-olds may show improved learning of the word-object associations relative to the action-object associations. Furthermore, we predicted that this word benefit ought to increase with age, especially around the 24-month mark, with children on the other side of the vocabulary spurt, who may show increased sensitivity to the associations between words and objects. By 36 months of age, owing to increased cognitive resources, we anticipated that children (and indeed adults) learn both word-object and action-object associations with similar ease. We made the decision to include the 36-month-olds based on the results of the 12- and 24-month-olds.

## Methods

### Participants

Thirty-four German monolingual 12-month-olds (range = 11;44—12;59 months; mean = 12;02, female = 14), thirty-six 24-month-olds (range = 22;39—25;78 months; mean = 24;19, female = 17), thirty-nine 36-month-olds (range = 34;23—37;84 months; mean = 35;84, female = 17) and 27 adults (range = 19;06—28;71 years; mean = 22;44, female = 19) participated in the experiment. Fourty-eight additional participants were tested but excluded from the analysis because of piloting (5), unwillingness to participate (2), technical failure (5), own cat was called “Loki” which resembles one of the novel words (1), bilingualism (3), preterm birth (1), or insufficient data (31, see Preprocessing). Thus, exclusion was overall at 26.1%, with 16.9% for data reasons and 11.5% for any other reasons. Children were recruited from the babylab database and participation was rewarded with a book. Caregivers of 12-month-olds completed the ELFRA-1 (Elternfragebögen für die Früherkennung von Risikokindern; [24]), while caregivers of the 24-month-olds completed the German adaption of the FRAKIS (Fragebogen zur frühkindlichen Sprachentwicklung; [25]) in order to control for children’s language abilities. Additionally, cognitive and fine motor tests using the Bayley Scales of Infant and Toddler Development were administered for both age groups in the lab [26]. Parents signed a written informed consent form for their child. Adults were mostly students of the University, also signed the written consent form, and were rewarded with either 0.5 course credit points or € 4. Ethics approval was granted by the ethics committee of the Georg-Elias-Müller-Institute for Psychology, University of Goettingen (Project 123).

### Stimuli

**Fig 1:**
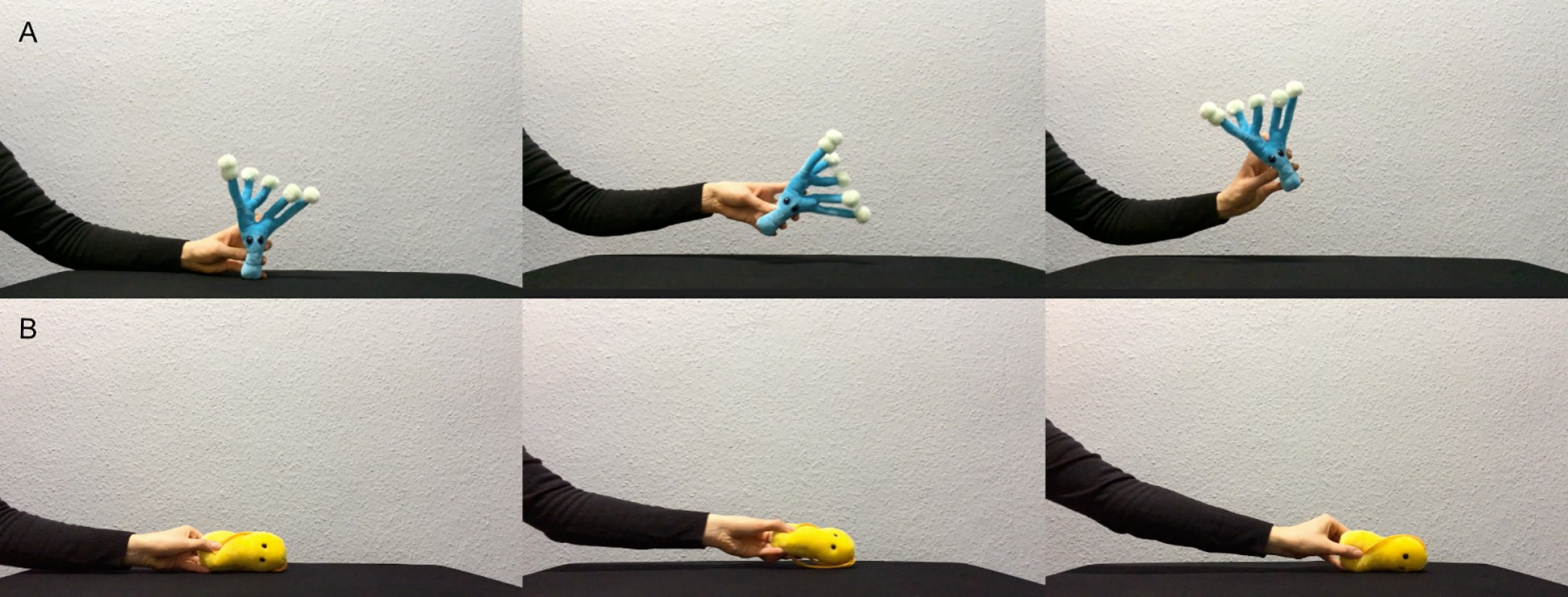
Stimuli. Blue and yellow germ toys were used as novel objects. As novel actions, an upward movement with tilting to the sides, and a sideways movement with tilting backwards and forwards were used.

We selected two novel words in keeping with the phonotactic rules of German (Tanu and Löki), two arbitrary actions (described in further detail below), and two novel objects (a yellow and a blue soft toy germ from https://www.giantmicrobes.com/us/, see Fig 1). Audio stimuli were recorded by a female German native speaker in infant-directed speech. The labels were embedded in separate carrier phrases for the training (e.g., “Schau mal, ein Tanu!” meaning “Look, a Tanu!”) and test phase (e.g., “Hey, guck mal, wo ist denn das Tanu?”, meaning “Hey, look, where is the Tanu?”).

#### Training phase stimuli

Training phase stimuli presented children with both word-object and action-object associations during the course of each trial. The visual stimuli presented a hand (with the arm of the agent being visible) moving one of the objects according to two selected actions. When moving up and down (Fig 1, panel A), the hand began on the lower middle of the screen and slowly moved upwards and then down again while slightly leaning to the right and to the left in an alternating manner. When moving sideways (Fig 1, panel B), the action would begin in the middle of the screen and the object would slightly turn backwards and forwards while moving first to one side of the screen, then backwards to the other, and ending up in the middle of the screen again, and both actions were roughly 7 s long. We chose these actions in preference to some previously selected actions with younger infants (e.g., shaking and looming) since these previously selected actions might be used to draw attention to the object and need not necessarily be considered object-related actions.

Auditory stimuli were presented before the onset of the action in half of the trials, and after the action had ended in the other half of the trials to control for influences of order of presentation of word-object and action-object associations. Each video in the training phase was ten seconds long, 720x420 pixels in size, and presented in the middle of the screen.

#### Test phase stimuli

The stimuli for the test phase included baseline stimuli, prime stimuli and recognition stimuli. Baseline and recognition stimuli were identical and involved children being presented with stationery images of the two objects in silence. Images (baseline and recognition) were 640 x 480 pixels and appeared next to each other in the center of the left and right half of the screen. Prime stimuli involved children hearing either the label for one of the objects or videos of the action movements described above, although the actions were now performed in the absence of the object. This was to ensure that the action-object association was not prioritized in any way and kept as similar to the word-object prime phase as possible (including the absence of a referent). Seeing the action being performed on the object would cue children to the identity of the correct object mapping. We also wanted to avoid using a novel control object because it could be considered as a new reference object. The videos of the action movements lasted seven seconds and were presented in the center of the screen. The label for the target object was embedded in a carrier phrase that similarly lasted seven seconds, with the label occurring at the end of the phrase.

### Procedure

Participants sat in a dimly lit and quiet experimental room at a distance of 65 cm from a TV screen (92 x 50 cm), where the visual stimuli were presented. A remote eye tracker (Tobii X 120), set on a platform underneath the TV screen, was used to record gaze data at 60 Hz. Stimuli were presented using E-Prime software. Auditory stimuli were presented through two loudspeakers situated above the television screen. Further, two video cameras centered above the screen served to monitor the participant during the experiment. Prior to testing, gaze was calibrated using a 5-point calibration procedure, in which a red point appeared in every corner and the center of the screen. The experiment only started following successful calibration. Each trial began with a Teletubby serving as a fixation getter in the middle of the screen against a black background, followed by the stimulus presentation.

After the eye-tracking part, the cognitive and finemotor scales of the Bayley scale were administered with the 12- and 24-month-olds [26]. This was part of a larger cross-laboratory project to examine the correlation between performance in these scales across development. These data and the vocabulary data will not be reported here.

### Experimental design

Each child was presented with a training phase and a test phase (see Table 1). We ensured that word-object and action-object associations were counterbalanced across children resulting in four different training lists with each of the objects being associated with each of the labels and each of the two actions, and according test phases. Presentation order of all trials within the individual training and test phases was fully randomized.

**Table 1:** Experimental design. Overview of Learning and Test phase. The order of trials within one phase was fully randomized.

Phase	N trials	Description
Learning phase	8	2 novel objects (à 4 trials) with distinct novel label and novel action
Test phase (critical)	8	4 word-object test trials and 4 action-object test trials
Test phase (control)	4	word-action test trials

#### Training phase

This phase consisted of eight trials where children saw individual objects onscreen with four trials per object. In each trial, children were presented with one object and heard a distinct label for this object and saw a distinct action being performed on the object. Each trial presented both the word-object and the action-object association, with half of the trials presenting the word-object association first and the other half of the trials presenting the action-object association first. This is in contrast to studies that ensured temporal synchronicity between word and action presentations (e.g., [27, 28]). In our study, we deliberately avoided temporal synchronicity for two reasons. First, this would help to ensure that children did not associate the word with the action (similar to the literature on word-action learning, i.e., verb learning). Second, we wanted to give children time to process each association separately, which has been shown to support learning [29].

#### Test phase

Each test trial was divided into a baseline phase, a prime phase and a recognition phase. During the baseline phase, children saw both static objects side-by-side on screen in silence for 2.5 s. The objects disappeared after 2.5 s. Next, in the prime phase of 7 seconds (similar to [30]), children either heard the label for one of the objects (word-object trial) or saw a hand performing one of the actions (action-object trial), in the absence of the object. The recognition phase began 300 ms after the offset of the label or end of the action, at which point the objects reappeared on-screen as static pictures once again for 2.5 s in the same positions as in the baseline phase. Across eight test trials, children were tested equally often on their knowledge of the word-object and action-object associations (four trials each) for both objects.

Following these eight critical trials, we examined whether participants had associated the words with the actions presented (word-action trial) across four trials. We included these control trials to examine the possibility that participants had formed a word-action association that may influence responding in our critical test of word-object and action-object association learning. Here, the actions were presented side-by-side in silence (baseline phase) for 6500 ms. Then, in a prime phase, participants were presented with a black screen and heard one of the labels. 300 ms after the offset of the label. During the recognition phase, the actions reappeared on-screen in the same positions for further 6500 ms.

Note that both word-object and action-object association types were tested as a within-subject factor, and we refer to these as “conditions” in the rest of the paper.

### Pre-processing

The eye-tracker provided an estimate of where participants were fixating in each time stamp during the trial, with one data point approximately every 16 ms. All data (gaze data and trial information) were exported from E-Prime and then further processed in R (R version 3.2.4 (2016-03-10), [31]). For each time stamp, data were only included when one or both eyes of the participant were tracked reliably (validity less than 2 on E-Prime scale). When both eyes were tracked, the mean gaze point for both eyes was computed for further analysis. Gaze data were then aggregated into 40 ms bins. Two areas of interest covering the location and size of the two images presented on-screen respectively were defined based on the location and size of the two images on-screen in the test phase.

For the baseline and recognition phase, we coded whether the participant looked at the correct object (i.e., the target), the distractor or at neither of these onscreen for each timepoint. Typically, fixation data is analyzed from 240 ms to 2000 ms from the onset of a label [32], since only these fixations can be reliably construed as a response to an auditory stimulus. Following this convention, we analyzed only data from 240 ms from the onset of the baseline phase and the recognition phase. This ensured that we avoided early fluctuations in fixations as a result of the images appearing on-screen. Note that this is in addition to the 300 ms between the offset of the label and the action at the end of the prime phase, thereby allowing participants adequate time to process the information provided.

Across word-object, action-object and word-action trials, we corrected the recognition phase for any fixations to either object in the baseline phase on an individual trial level (i.e., each time point in the recognition phase was corrected for the overall looking score in the baseline phase on that particular trial). This baseline-corrected measure was our dependent variable in all analyses.

In order to ensure that we analyzed similar durations of the word-action and word-object and action-object trials, we only analyzed 2000 ms of the recognition phase of the word-action trials. Since we wanted to use target looking in the word-action condition as a control variable in analyzing participants’ learning of word-object and action-object associations, we calculated the difference in preferential target looking from the baseline to the recognition phase in word-action trials and included this value as an index of the formation of word-action associations as a control variable.

Single test trials were excluded if a participant was looking at the stimuli less than M − 3SD of the time. Thus, a trial was excluded if a 12-month-old child looked less than 8.4% of the time during the trial to one of the two pictures. This led to an exclusion of 27 trials (5.24%). For 24-month-olds, this criterion was at ≤ 30.3%, 26 trials (5.67%); for 36-month-olds it was at ≤ 34%, 34 trials (6.35%); and for adults at ≤ 50%, 11 trials (3.13%). This led to one further exclusion as one participant did not look enough during any of their trials (24: N = 1). Furthermore, participants were excluded from the analyses if the participant contributed only one trial per condition in the test phase (12: N = 8; 24: N = 5; 36: N = 5; Adults: N = 3). Further, nine participants were missing data in either the baseline or the recognition phase in one of the conditions (12: N = 5, 36: N = 3, Adults: N = 1). Together, this led to exclusions of 13 12-month-olds, six 24-month-olds, eight 36-month-olds, and four adults, and left us with 136 included participants in the final sample.

## Data Analysis

Within each age group, we report two statistical approaches here. First, we report traditional t-tests and correlations. In particular, we ran a paired samples t-test comparing the proportion of target looking in the word-object and the action-object trials. Then we ran separate one sample t-tests comparing the baseline-corrected proportion of fixations to the target during the recognition phase against chance (0 = no difference between baseline and recognition phase). Next, we report generalized linear mixed models. These models have two advantages over traditional analyses. First, they allow us to control for variability along additional dimensions, such as object, label, action, and performance in word-action trials. Second, they also allow us to include the factor Time during the trial, thereby examining looking patterns over the whole time course of the trial (for a more detailed description and instruction see [33, 34]).

**Fig 2:**
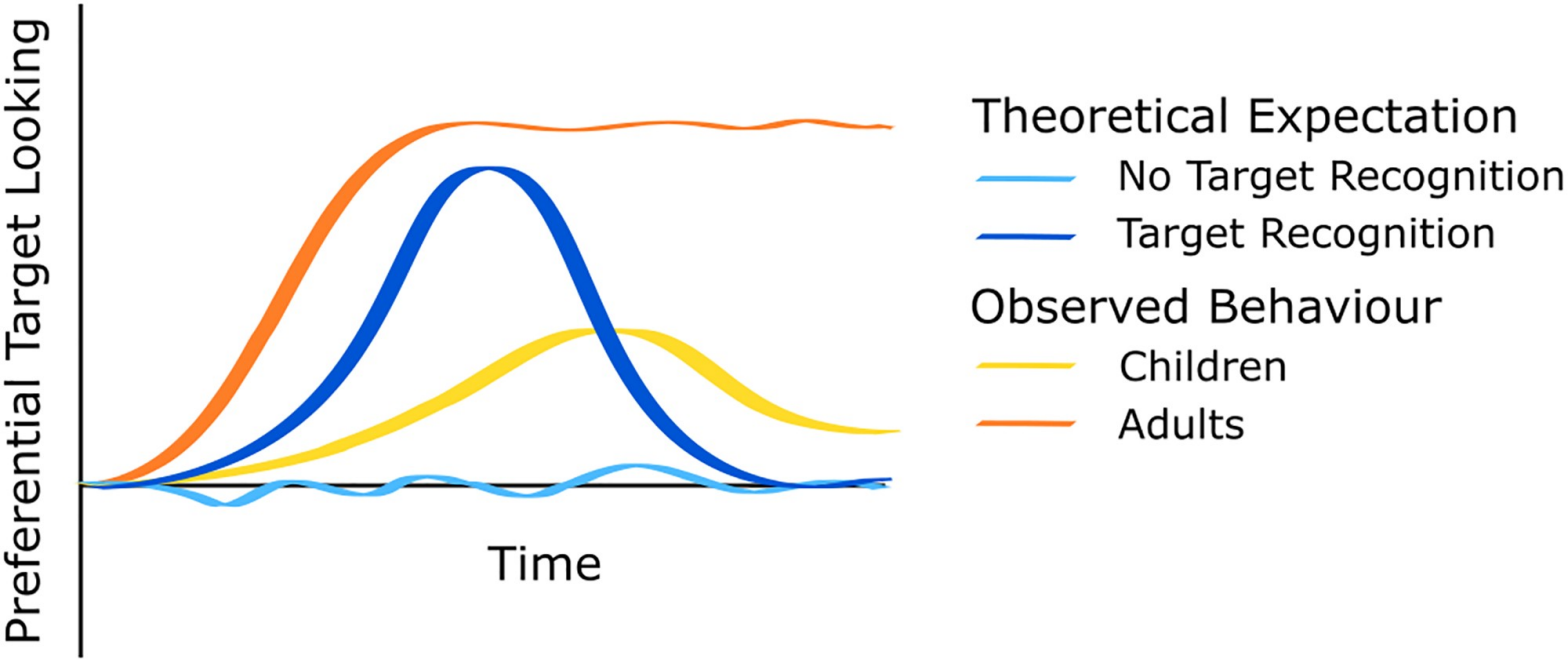
Streamer plot of theoretical expectations and typical behavioral observations of looking behavior across time. Light and dark blue represent theoretical expectations, yellow and orange represent typical behavior.

We included Time and its second, third, and fourth polynomial in the model. This allows us to model our data as a linear, quadratic, cubic, and quartic function of time. In Fig 2, we plotted theoretically expected and typically observed curvatures of target recognition. If participants did not learn the tested associations, and therefore, do not show target recognition at test, they typically look back and forth between the two objects, meaning that their target looking is around chance level (pictured in light blue) throughout the trial. If participants have learned the association, and therefore show target recognition at test, their target looking would be first at chance level, would later increase when they look to the correct object, and goes back to chance level (pictured in dark blue). This would be reflected in a quadratic curve, or a quartic curve when tails are included at the beginning and end. In practice, adults usually look at the correct object until the end of the trial when they have learned the correct association (pictured in orange), thereby showing their recognition of the target [33]. This curve is often better fitted with a linear (steep rise) or cubic function (steep rise and high plateau). Children’s responses are often weaker and increases in target looking might start slightly later (pictured in yellow). However, their responses are also typically more variable requiring greater flexibility in model fitting and interpretation.

We fitted a generalized mixed model (GLMM) using lme4’s lmer function in R [35] with Gaussian error structure and identity link function. Condition and Time, and their interaction were included as fixed effects of interest. Further, Object, Name, and Action, as well as the baseline-corrected proportion of target looking in the word-action condition were included as fixed effects. We note we included performance in the word-action control trials in the model to ensure that performance in the word-object and action-object conditions was not driven by spurious associations being formed between words and actions. We also included Participant id and Condition as random factors to allow for random slopes across participants. The reduced model did not include Condition. A comparison between the reduced model and the full model including the factor Condition then allowed us to evaluate the influence of the factor Condition (action-object associations vs. word-object associations). We used the function drop1 to evaluate the influence of each factor in the model. This function compares the model including one factor with a model without this factor, and thereby evaluates its contribution to the model.

Visual inspection of a qq-plot and a histogram of the residuals showed a normal distribution, but, as is usually the case with looking time data, homogeneity seemed to be violated when plotting residuals against fitted values. Log-transforming the response did not contribute to an improvement of the model. Therefore, we chose the first model but results need to be reviewed with care.

For ease of interpretation and due to the complex interactions between conditions across the four age groups found in an omnibus ANOVA and GLMM, we do not report these analyses here. The results of these analyses can be found on osf.io/b7fmt. Here, we report only analyses split by age group to better capture the learning effects within each age group.

## Results

**Fig 3:**
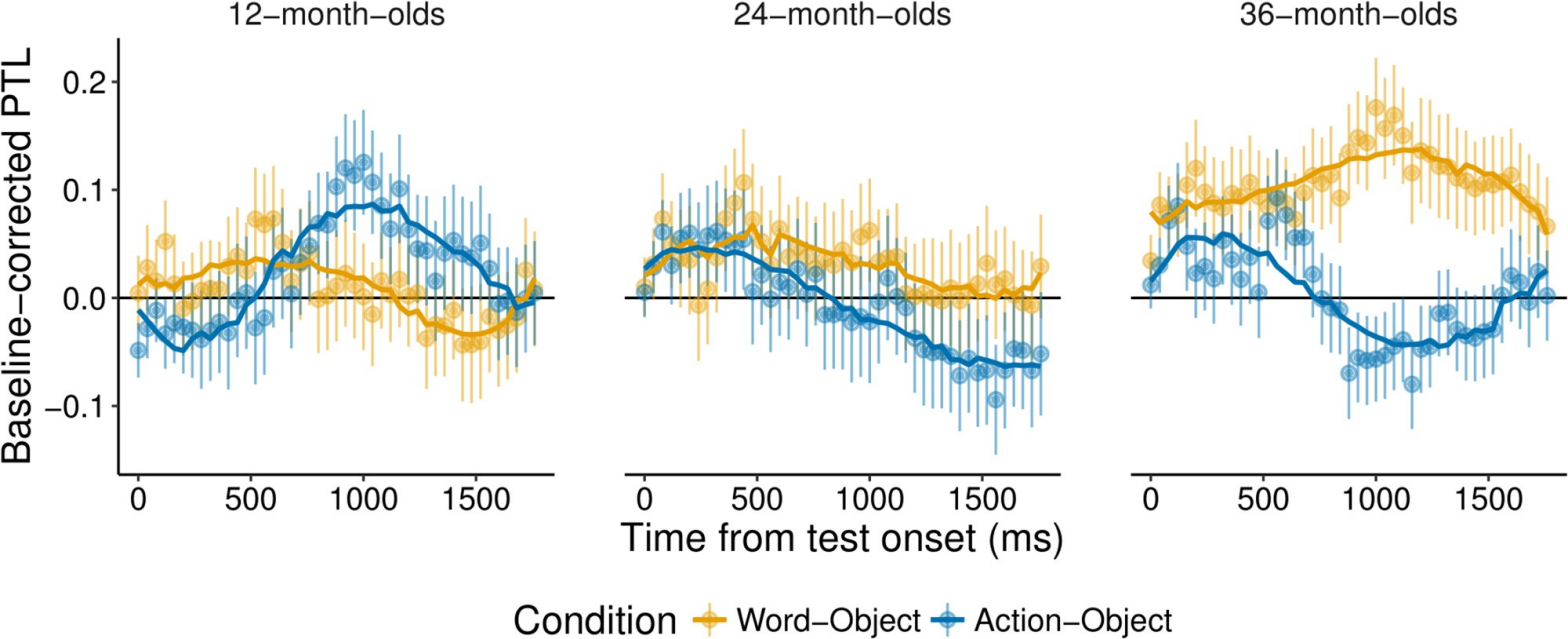
Time course of children’s target looking. Time course graphs for each of the children’s age groups of baseline-corrected preferential target looking (PTL) during the recognition phase. The word-object condition is represented in yellow, and the action-object condition in blue. The line at 0 represents chance level (increase from baseline), fixations above this line indicate target looking whereas fixations below this line indicate distractor looking. The first 240 ms are cut to allow for fixation time, and the time within the trial has been corrected, so that the x-axis starts at 0.

**Fig 4:**
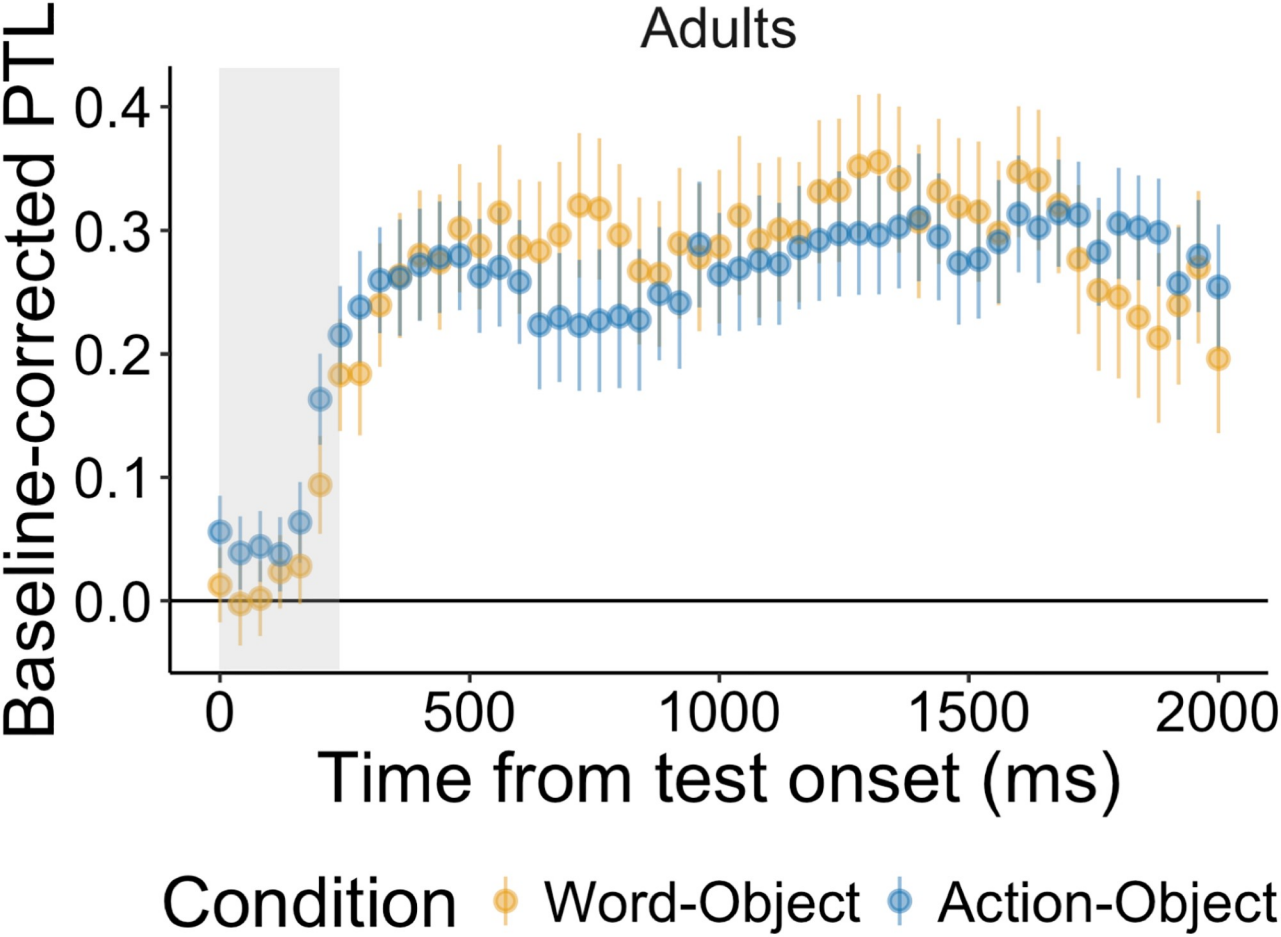
Time course of adults’ target looking. Time course graphs for the adults’ baseline-corrected preferential target looking (PTL) during the recognition phase. The word-object condition is represented in yellow, and the action-object condition in blue. The line at 0 represents chance level (increase from baseline), increases indicate target looking whereas decreases indicate distractor looking. Note that adults’ target looking starts already at the beginning of the trial. Nonetheless, we discarded the first 240 ms of the trial to match the children’s analyses.

Descriptives for each condition per age group can be seen in Table 2. An overview of the results can be seen in Tables 3 and 4, and in Figs 3 and 4.

**Table 2:** Descriptives. Descriptives of baseline-corrected preferential target looking in the recognition phase of the word-object and the action-object condition per age group (12, 24, 36 months, and adults). Values higher than 0 indicate target looking, values below 0 indicate distractor looking.

Age group	Condition			
	word-object		action-object	
	Mean	SD	Mean	SD
12-month-olds	.01	.16	.02	.13
24-month-olds	.03	.17	-.004	.15
36-month-olds	.1	.15	.003	.1
Adults	.26	.29	.25	.19

**Table 3:** Generalized linear mixed models analyzing the effect of condition for each age group to model baseline-corrected preferential target looking over time, including time and and its linear, quadratic, cubic and quartic term. res <- lmer(PTL_corr.mean ∼ Condition*(poly1+poly2+poly3+poly4) + Object + Name + Action + WA + (poly1+poly2 || id) + (poly1+poly2 | id:Condition), data = d.adults‥gca, REML = F, control = contr).

Group	Factor	Estimates	*SE*	lower CI	upper CI	LRT	*p*
12 months	Intercept	-0.01	0.03	-0.06	0.04		
	Object	0.01	0.01	-0.01	0.02	0.85	0.36
	Name	0.04	0.01	0.02	0.06	21.13	**<0.001**
	Action	0.04	0.01	0.02	0.06	20.44	**<0.001**
	WA	0.13	0.10	-0.07	0.33	1.60	0.21
	poly1:Condition	-0.26	0.20	-0.63	0.12	1.72	0.19
	poly2:Condition	0.22	0.12	-0.00	0.46	3.30	0.07
	poly3:Condition	0.14	0.06	0.02	0.25	5.58	**0.02**
	poly4:Condition	-0.04	0.06	-0.16	0.07	0.56	0.45
24 months	Intercept	0.01	0.03	-0.05	0.07		
	Object	-0.01	0.01	-0.03	0.01	0.72	0.40
	Name	-0.02	0.01	-0.04	-0.01	6.38	**0.01**
	Action	-0.00	0.01	-0.02	0.02	0.02	0.90
	WA	-0.02	0.10	-0.21	0.18	0.04	0.83
	poly1:Condition	0.22	0.20	-0.16	0.64	1.21	0.27
	poly2:Condition	-0.02	0.13	-0.26	0.27	0.01	0.91
	poly3:Condition	0.02	0.06	-0.10	0.14	0.18	0.67
	poly4:Condition	0.03	0.06	-0.08	0.15	0.29	0.59
36 months	Intercept	0.03	0.02	-0.01	0.07		
	Object	0.03	0.01	0.02	0.05	20.12	**<0.001**
	Name	-0.11	0.01	-0.12	-0.09	206.74	**<0.001**
	Action	0.02	0.01	0.01	0.04	10.55	**<0.001**
	WA	0.03	0.06	-0.10	0.14	0.19	0.66
	poly1:Condition	0.17	0.20	-0.25	0.53	0.68	0.41
	poly2:Condition	-0.21	0.12	-0.44	0.04	2.97	0.08
	poly3:Condition	-0.23	0.05	-0.32	-0.13	20.59	**<0.001**
	poly4:Condition	0.04	0.05	-0.05	0.14	0.77	0.38
Adults	Intercept	0.17	0.05	0.06	0.26		
	Object	-0.07	0.01	-0.08	-0.05	66.96	**<0.001**
	Name	-0.00	0.01	-0.02	0.02	0.02	0.90
	Action	0.03	0.01	0.02	0.05	17.55	**<0.001**
	WA	0.41	0.09	0.22	0.59	14.57	**<0.001**
	poly1:Condition	-0.14	0.15	-0.43	0.15	0.76	0.38
	poly2:Condition	-0.19	0.14	-0.46	0.09	1.78	0.18
	poly3:Condition	0.08	0.05	-0.03	0.18	2.18	0.14
	poly4:Condition	-0.03	0.05	-0.13	0.07	0.43	0.51

**Table 4:** Generalized linear mixed models for word and action conditions for each age group separately to model baseline-corrected preferential target looking over time, including time and and its linear, quadratic, cubic and quartic term. res ¡- lmer(PTL_corr.mean ∼ (poly1+poly2+poly3+poly4) + Object + Name + Action + WA + (poly1+poly2 || id), data = d.adults.word.gca, REML = F, control = contr).

Group	Factor	Estimates	*SE*	lower CI	upper CI	LRT	*p*
12 months: action	Intercept	-0.05	0.02	-0.10	-0.00		
	poly1	0.14	0.15	-0.14	0.44	0.86	0.35
	poly2	-0.24	0.10	-0.44	-0.03	4.89	**0.03**
	poly3	-0.07	0.04	-0.16	0.00	3.20	0.07
	poly4	0.11	0.04	0.03	0.19	6.61	**0.01**
	Object	0.06	0.01	0.03	0.08	18.29	**<0.001**
	Name	0.06	0.01	0.04	0.09	24.19	**<0.001**
	Action	0.04	0.01	0.01	0.06	9.35	**<0.001**
	WA	-0.07	0.13	-0.33	0.18	0.34	0.56
12 months: words	Intercept	0.02	0.03	-0.04	0.08		
	poly1	-0.14	0.13	-0.40	0.11	1.13	0.29
	poly2	-0.01	0.10	-0.20	0.18	0.01	0.92
	poly3	0.06	0.04	-0.02	0.14	2.18	0.14
	poly4	0.07	0.04	-0.02	0.15	2.63	0.10
	Object	-0.04	0.01	-0.06	-0.02	9.95	**<0.001**
	Name	0.01	0.01	-0.01	0.04	1.19	0.28
	Action	0.04	0.01	0.02	0.06	10.78	**<0.001**
	WA	0.32	0.16	0.01	0.63	3.80	**0.05**
24 months: actions	Intercept	0.04	0.03	-0.02	0.09		
	poly1	-0.29	0.14	-0.56	-0.01	4.11	**0.04**
	poly2	-0.07	0.11	-0.27	0.14	0.43	0.51
	poly3	0.05	0.04	-0.02	0.14	1.72	0.19
	poly4	-0.02	0.04	-0.09	0.06	0.16	0.69
	Object	-0.03	0.01	-0.06	-0.01	6.96	**0.01**
	Name	-0.05	0.01	-0.08	-0.03	15.93	**<0.001**
	Action	-0.00	0.01	-0.03	0.02	0.14	0.70
	WA	-0.06	0.14	-0.32	0.19	0.21	0.65
24 months: words	Intercept	0.02	0.03	-0.05	0.08		
	poly1	-0.06	0.15	-0.34	0.24	0.15	0.70
	poly2	-0.10	0.10	-0.31	0.09	0.99	0.32
	poly3	0.08	0.04	0.00	0.16	4.04	**0.04**
	poly4	0.01	0.04	-0.07	0.09	0.09	0.76
	Object	0.02	0.01	-0.01	0.04	2.15	0.14
	Name	0.00	0.01	-0.02	0.03	0.16	0.69
	Action	0.01	0.01	-0.02	0.03	0.20	0.65
	WA	-0.06	0.16	-0.37	0.24	0.16	0.69
36 months: actions	Intercept	0.01	0.02	-0.02	0.05		
	poly1	-0.15	0.14	-0.42	0.12	1.20	0.27
	poly2	0.11	0.09	-0.06	0.29	1.46	0.23
	poly3	0.14	0.03	0.07	0.20	15.20	**<0.001**
	poly4	-0.04	0.03	-0.11	0.03	1.18	0.28
	Object	0.01	0.01	-0.00	0.03	2.03	0.15
	Name	-0.09	0.01	-0.11	-0.07	78.43	**<0.001**
	Action	0.05	0.01	0.03	0.07	25.41	**<0.001**
	WA	0.03	0.07	-0.11	0.18	0.20	0.66
36 months: words	Intercept	0.15	0.03	0.10	0.20		
	poly1	0.01	0.15	-0.28	0.29	0.01	0.94
	poly2	-0.10	0.09	-0.28	0.07	1.36	0.24
	poly3	-0.09	0.04	-0.16	-0.02	6.68	**0.01**
	poly4	0.01	0.04	-0.06	0.07	0.04	0.85
	Object	0.06	0.01	0.03	0.08	27.53	**<0.001**
	Name	-0.13	0.01	-0.15	-0.11	139.21	**<0.001**
	Action	-0.01	0.01	-0.03	0.01	0.49	0.49
	WA	0.05	0.11	-0.17	0.27	0.20	0.66
Adults: actions	Intercept	0.12	0.05	0.04	0.22		
	poly1	0.12	0.13	-0.14	0.37	0.84	0.36
	poly2	-0.02	0.11	-0.25	0.20	0.04	0.85
	poly3	-0.08	0.04	-0.14	-0.01	4.55	**0.03**
	poly4	-0.04	0.04	-0.11	0.03	1.18	0.28
	Object	0.05	0.01	0.03	0.07	20.80	**<0.001**
	Name	-0.01	0.01	-0.03	0.01	1.37	0.24
	Action	0.04	0.01	0.02	0.06	15.51	**<0.001**
	WA	0.36	0.11	0.16	0.57	9.29	**<0.001**
Adults: words	Intercept	0.22	0.07	0.08	0.36		
	poly1	-0.02	0.10	-0.20	0.17	0.03	0.86
	poly2	-0.20	0.08	-0.37	-0.03	5.54	**0.02**
	poly3	-0.00	0.04	-0.07	0.07	0.01	0.93
	poly4	-0.07	0.04	-0.14	0.00	3.86	**0.05**
	Object	-0.18	0.01	-0.20	-0.16	251.71	**<0.001**
	Name	0.01	0.01	-0.01	0.03	1.14	0.29
	Action	0.02	0.01	0.00	0.05	4.82	**0.03**
	WA	0.47	0.17	0.16	0.81	7.07	**0.01**

### 12-month-olds

#### T-tests

For the 12-month-olds, a paired samples t-test comparing word-object and action-object conditions found no significant difference between conditions, *p* = .52. Separate one sample t-tests comparing baseline-corrected target fixations in each of the conditions to chance (chance = 0) were not significant (all *p*s >.1).

#### GLMM

For the 12-month-olds, the model comparison between the full model including condition (word-object vs. action-object) and the reduced model was significant (*χ*2 = 15.31, df = 5, *p* = .009). Using drop1, the model revealed a significant interaction of Condition*poly3 (*χ*2 = 5.58, df = 1, *p* = .018). The interaction of Condition*poly2 was at *χ*2 = 3.30, df = 1, *p* = .069. Thus, the model revealed differences mainly in the cubic curvature across conditions. This captures differences in two peaks in the time course of fixations across the two conditions, hence necessitating the cubic curve. As Fig 3 shows, fixations in the word-object condition show a slight bump early in the trial flattening out towards the end of the trial, while fixations in the action-object condition show a later bump from around 500 ms with an increase in fixations to the target in this condition relative to the word-object condition.

For the model, examining the data of the word-object trials alone (word-object split model), none of the time terms were significant. The influence of performance in the word-action control trials on target fixations in the word-object condition was at *χ*2 = 3.8, df = 1, *p* = .051 (a simple correlation confirmed this effect with performance in the word-object condition positively correlating with performance in the word-action trials, rho (Spearman) = .4, *p* = .018).

For the model, examining the data of the action-object trials alone (action-object split model), the quadratic time term (*χ*2 = 4.89, df = 1, *p* = .027), and the quartic term were significant (*χ*2 = 6.61, df = 1, *p* = .01). As indicated in Fig 3, 12-month-olds showed an increase in target fixations around 500 ms into the trial, captured by the quadratic curve, while the quartic curve captures two changes in the curve of fixations, with an early slight dip in target looking at the beginning of the trial and a later stronger increase to the target around 500 ms. Such a pattern of looking is in keeping with our theoretical expectations of target looking behaviour, with a classic quadratic curve when only looking at the later part of the trial and a slower increase towards target looking early in the trial.

In summary, for 12-month-olds, we find evidence for differences in looking behaviour in the test phase (from the baseline phase) in the action-object condition. Such a pattern of looking behaviour is typically been interpreted as evidence of target recognition (see our discussion of expected patterns above). These differences in target fixations were not significant across the entire time course of the trial, as indicated by the t-tests reported, highlighting potential concerns with the robustness of this effect at this age.

### 24-month-olds

#### T-tests

For the 24-month-olds, a paired samples t-test comparing word-object and action-object conditions found no significant difference between conditions, *p* = .37. Separate one sample t-tests comparing baseline-corrected target fixations in each of the conditions to chance (chance = 0) were not significant (all *p*s >.1).

#### GLMM

For the 24-month-olds, the model comparison between the full model including condition (word-object vs. action-object) and the reduced model was not significant (*χ*2 = 2.83, df = 5, *p* = .73). Using drop1, the model revealed no interaction between Condition and any of the time terms.

To summarize, at 24-months, we did not find robust evidence for action-object learning nor for word-object learning, and we did not find a difference in target recognition across conditions (in both analyses, t-tests and GLMM).

For consistency with the other age groups, we split the data from the word-object and action-object trials. For the word-object split model, the cubic time term was significant (*χ*2 = 4.04, df = 1, *p* = .044), suggesting that 24-month-olds showed increased looks to the target object (followed by a decline) relative to baseline. For the action-object split model, the linear time term was significant (*χ*2 = 4.11, df = 1, *p* = .043) which reflected a slow decrease in target looking over time (reduced relative to baseline) and does not match theoretical expectations with regard to the curvature associated with target recognition. Given issues with the interpretation of this result against the background of non-significant model comparison, we report these results here for thoroughness but will not interpret this as evidence of learning until further data corroborates this finding.

### 36-month-olds

#### T-tests

For the 36-month-olds, a paired samples t-test revealed significant differences between word-object and action-object conditions (t(38) = -3.39, *p* = .002, d = -0.77). Separate one sample t-tests comparing baseline-corrected target fixations in each of the conditions to chance (chance = 0) found a significant difference from chance in the word-object (t(38) = 4.27, *p* <.001, d = 0.97) but not the action-object condition (t(38) = 0.12, *p* = .9, d = 0.03). These results suggest that 36-month-olds learned word associations but not action associations for objects.

#### GLMM

For the 36-month-olds, the model comparison between the full model including Condition (word-object vs. action-object) and the reduced model was significant (*χ*2 = 36.34, df = 5, *p* <.001). Using drop1, the model revealed a significant interaction of Condition and the cubic time term (*χ*2 = 20.59, df = 1, *p* <.001), suggesting that there were differences across conditions.

For the word-object split model, the cubic time term was significant (*χ*2 = 6.68, df = 1, *p* = .01). Also for the action-object split model, the cubic time term was significant (*χ*2 = 15.20, df = 1, *p* <.001). As is evident in Fig 3, these results suggest that 36 month-olds fixate the target consistently across the recognition phase of the trial (more than at baseline) in word-object trials. In contrast, in action-object trials, they display a brief period of initial target recognition fixating the target above chance (increase from baseline) in the recognition phase, followed by fixations towards the distractor and again towards the target.

To summarize, 36-month-olds showed systematic looking patterns in both the word-object and the action-object condition, although the effects of target recognition were more in keeping with theoretical expectations for the word-object condition compared to the action-object condition. Taken together with the results of the t-tests, we interpret these results as being indicative of word-object association learning but not action-object association learning at 36 months.

### Adults

#### T-tests

For the adults, a paired samples t-test comparing word-object and action-object conditions found no significant difference between conditions, *p* = .83. Separate one sample t-tests comparing baseline-corrected target fixations in each of the conditions to chance (chance = 0) revealed significant differences from chance in both conditions (word-object: t(26) = 4.85, *p* <.001, d = 1.32; action-object: t(26) = 6.91, *p* <.001, d = 1.88), suggesting that they formed both word-object and action-object associations.

#### GLMM

For the adults, the model comparison between the full model including condition (word-object vs. action-object) and the reduced model was not significant (*χ*2 = 4.72, df = 5, *p* = .45). Using drop1, the model revealed no significant interaction of Condition with any of the time terms. Thus, there were no significant differences between conditions.

For the word-object split model, the quadratic term (*χ*2 = 5.54, df = 1, *p* = .019) and the quartic time term were significant (*χ*2 = 3.86, df = 1, *p* = .049). Furthermore, there was an influence of performance in the word-action condition on looking times in the word-object condition (*χ*2 = 7.07, df = 1, *p* = .008). For the action-object split model, the cubic time term was significant (*χ*2 = 4.55, df = 1, *p* = .033). Furthermore, there was a significant influence of performance in the word-action condition on looking times in the action-object condition (*χ*2 = 9.29, df = 1, *p* = .002), confirmed in a correlation analysis, (rho (Spearman) = .58, *p* = .002). Importantly, both analyses controlled for performance in the word-action control trials when examining the effect of learning of word-object and action-object associations.

To summarize, adults learned the associations in both word-object and action-object conditions.

## Discussion

In the current study, we explored the developmental trajectory of children learning to associate words and actions with objects. In particular, we asked, whether there are differences across development and across information types in the learning of such associations when children are given the opportunity to learn both a word and an action association for an object. In a first training phase, participants were familiarized with novel objects that were associated with both a novel label and a novel action. In the following test phase, participants’ learning of word-object and action-object associations was tested by measuring their looks to the target object following presentation of either the associated action or the associated word. 12-month-olds showed some evidence of learning of action-object associations but not word-object associations when considering time within the trial, 24-month-olds learned neither word-object nor action-object associations (and there was no evidence for a difference in performance across conditions), 36-month-olds also showed learning of word-object associations but no systematic learning of action-object associations, and adults learned both word-object and action-object associations. These results plot the developmental trajectory in children and adults’ learning of word-object and action-object associations.

Our results are in keeping with the developmental pattern that has been observed in the related field of iconic gestural learning in typically developing children. Namy, Campbell, and Tomasello compared children’s learning of iconic (a hopping rabbit) and arbitrary (side-to-side motion) gestures across early childhood and found that 18-month-olds and 4-year-olds successfully mapped both types of gestures with the associated objects, while 26-month-olds only learned to map iconic but not arbitrary gestures to the associated objects ([36], see also [37]). The authors argue that this U-shaped pattern emerges due to a reorganization of the gestural system at around two years of age, potentially due to an increased focus on linguistic communication from this age.

Our study extends these findings from iconic gestural learning to compare children’s learning of word-object and action-object associations across this malleable period of development. Our findings in action-object association learning are consistent with those reported by Namy and colleagues. 12-month-olds, like the 18-month-olds in [36], learnt only action-object associations, while 24-month-olds and 36-month-olds did not. Adults, like the 4-year-olds in [36], learned both word and action associations for objects, with no difference between the two conditions, suggesting a period of further flexibility in learning associations for objects past the age tested in [36]. Thus, as far as action-object associations are concerned, our results, together with [36], highlight a developmental pattern in early gesture-object and action-object association learning. Before exploring potential explanations for this developmental curve, we next discuss the results for the word-object condition in detail.

The results from the word-object condition differed considerably from the action-object condition, with younger children showing no evidence for word-object association learning. However, the literature on word learning suggests that even 12-month-olds have little difficulties learning words: infants understand words for objects (such as bodyparts) already at 6 months [9, 38]. Further, when presented with a novel word for a novel object, children as young as 6 to 12 months of age learn to associate words with objects ([23, 39], but see [40] that such learning is contingent on a number of factors, e.g., similarity of the two objects presented). Those studies, however, only presented infants with word-object associations and not both word- and action-object associations.

Studies presenting children with asynchronous input including multiple information types (as in the current study) indeed show different results. With younger children, studies presenting temporally synchronous multimodal input suggest that the temporal alignment of word and action may bolster word-object association learning at very early ages (6—8 months, [41]). Relatedly, studies presenting children with word associations for objects in motion, suggest that actions that are simultaneously presented, albeit not temporally aligned, with label information might help to highlight the connection between the word and the object, thereby boosting learning of word-object associations [42]. Nevertheless, Puccini and Liszkowski find that young infants (at 15 months of age) learned only word associations for objects (and not gesture-object associations), and only when word-object associations are presented without gestural information, potentially due to such gestures drawing attentional resources away from learning of word-object associations [19]. Thus, even 15-month-olds’ word learning is impacted by the presentation of different information types, with them learning word-object associations only when the stimuli are presented in the absence of such gestural information, at least when information from the gestural modality is asynchronous.

With regards to the results of the 12-month-olds, we found action-object association learning when we considered time within the trial, but not when we collapsed the data across time. This suggests, that learning in this condition could only be observed with high resolution, and therefore, might still be weak at this age. Nonetheless, the significant model comparison suggests that action-object association learning differed from word-object association learning, which we did not find in any of the analyses at this age.

Our results also highlight additional factors that may potentially influence word-object learning in 12-month-olds. We found a positive correlation between 12-month-olds’ performance in the word-object condition and the word-action condition. In other words, those 12-month-olds who mapped the words onto the actions performed on the objects that these words referred to (the word-action condition), also learned the word-object associations better. This raises the possibility that word-object association learning may be mediated by spurious associations between words and actions at this age, further highlighting the salience of actions at this age. Furthermore, it could be that 12-month-olds do not to choose between word-action and word-object associations for the same words, even though the words and the actions were not temporally aligned. Accordingly, it could be that 12-month-olds learned the word-object association, while at the same time maintaining action associations for this word (c.f. [43]), at the very least when the actions and words are not temporally synchronized. Such multiple associations might be restricted later in childhood, at two years of age, where we found no similar correlations between performance in the word-action and word-object condition, potentially due to children using morphosyntactic cues in the linguistic stimuli provided to map the words onto objects alone (and not actions; [44]).

Next, we discuss the findings from the 24-month-olds in further detail. Previous studies suggest that 24-month-olds learn words after minimal exposure (see [45] for an extensive review of early word learning). However, Hahn and Gershkoff-Stowe found that action-object associations were learned better than word-object associations at both 2- and 3-years of age ([17], see also [6], but see the results by [36, 37]). Our findings reveal no evidence for either action-object association learning nor word-object association learning at 24 months (given the absence of an influence of condition on target looking in the model comparison). We note that when we look at the time course of the 24-month-olds in both conditions, we see target looking at the beginning of the trial, but this target looking decreases over time, and therefore, does not mimic theoretically expected patterns. Together with the contrasting results in the literature to-date, it appears that both the gestural and the linguistic communication system at 24 months may be more susceptible to interference from other information types, especially when this information is presented simultaneously, albeit asynchronously. Indeed, this is in keeping with recent literature highlighting the influence of additional factors, e.g., language background, on word-object association learning at the same age [46].

By 3 years of age, we find systematic target looking in both the word-object and action-object condition, but found no modulation of the effect by performance in the word-action condition, similar to the 2-year-olds. However, we note that target looking in the action-object condition of the 36-month-olds followed a cubic curve with an initial increase towards the target, followed by distractor looking. In contrast, target looking in the word-object condition showed an early increase towards the target, which was sustained throughout the course of the trial. The difference in the time courses of these effects, and also when compared to theoretical assumptions (see Fig 2), might be taken to suggest that children did not learn action-object associations at this age, relative to word-object learning and action-object learning at older ages, e.g., at 4 years [14]. This is further evident in the non-significant result when comparing overall target looking against chance. Thus, for slightly younger children such as our 36-month-olds, verbal information might continue to receive more attention overall relative to action information.

Adults learned both word-object and action-object associations successfully, with no difference between conditions. Further, we found that performance in the action-object condition was positively correlated with performance in the word-action condition, raising the possibility that action-object associations in adulthood may be mediated by language, i.e., via the word-action and word-object mappings. However, we note that the GLMM found an influence of performance in the word-action condition on target fixations overall (across both conditions), potentially reflecting overall association strength for all three associations presented rather than specific modulation of performance in the action-object condition by performance in the word-action condition. Importantly, the inclusion of performance in the word-action control trials in the model revealed effects of word-object and action-object association recognition over and above potential effects of spurious associations between the words and actions presented.

Overall, our results suggest some evidence of early learning of action-object associations but not word-object associations, with increased learning of word-world mappings only later in childhood, given the physical and temporal attributes of the stimuli presented in the current study. Potentially, this shift to word-world mappings may occur due to children’s greater exposure to systematic one-to-one mappings between words and objects across the second and third year of life. Nevertheless, with increasing lexical exposure, children develop and might learn both word-object and action-object associations.

The early action learning effects call into question theories of language acquisition that highlight a special place for words in input-world mappings (e.g., [20]), and suggest rather that early mappings may be driven more by the salience of input available, with the potentially more salient action-object mappings taking precedence early in childhood. With time and increased exposure to word-world mappings, children may begin to attend more to the mapping between words and the world around them. This is further amplified with greater linguistic and cognitive competence, such that older children and adults learn to track word and action associations for the same objects.

Finally, we include a note on our statistical analysis. Although the results from the different statistical analyses diverged in some cases, we believe that the combination of t-tests and GLMMs allowed us to quantify our effects in a differentiated way. For example, the GLMMs suggested a quadratic curvature for 12-month-olds’ action learning. This pattern was not observable when target looking was averaged across time and t-tests were employed. These differences might suggest that target looking was rather brief and only observable when time within the trial was considered. Similarly, 36-month-olds’ target looking in the action-object condition manifested itself in a cubic curve with its form not being predicted by the theoretical assumptions. Here as well, the combination of t-tests and GLMMs proved valuable, highlighting the short-lived nature of 36-month-olds’ action-object association. Together, the combination of the different tests allowed us to better interpret our data and present a more differentiated picture of the development of early word and action learning.

Taken together with the previous literature on the topic, our findings suggest a distinct developmental pattern in children’s mapping of words to objects and actions to objects. We observed that children showed a preference for action-object associations early in life (at 12 months) and only later showed learning of word-object associations (at 36 months). It was much later in development, that we found some evidence for the maintenance of both action-object and word-object associations, leading to adults learning both word-object and action-object associations.

These results advance our understanding of children’s learning from action and word input in their environment: when presented with rich learning environments, selecting interesting or relevant aspects helps humans of all ages to structure the rich and multimodal input they perceive in the world.

## Supporting information

S1 Appendix (PDF)

## References

[1] May L, Byers-Heinlein K, Gervain J, Werker JF. Language and the newborn brain: does prenatal language experience shape the neonate neural response to speech? Frontiers in Psychology. 2011;2(222). 10.3389/fpsyg.2011.00222 21960980 PMC3177294

[2] Verbruggen SW, Kainz B, Shelmerdine SC, Hajnal JV, Rutherford MA, Arthurs OJ, et al Stresses and strains on the human fetal skeleton during development. Journal of the Royal Society Interface. 2018;15(138):20170593 10.1098/rsif.2017.0593 PMC5805961 29367236

[3] Gogate LJ, Bahrick LE, Watson JD. A study of multimodal motherese: The role of temporal synchrony between verbal labels and gestures. Child Development. 2000;71(4):878–894. 10.1111/1467-8624.00197 11016554

[4] Gogate LJ, Hollich G. Invariance detection within an interactive system: A perceptual gateway to language development. Psychological Review. 2010;117(2):496–516. 10.1037/a0019049 20438235

[5] Gogate LJ, Maganti M. The origins of verb learning: Preverbal and postverbal infants’ learning of word–action relations. Journal of Speech, Language, and Hearing Research. 2017;60(12):3538–3550. 10.1044/2017_JSLHR-L-17-0085 29143061

[6] Childers JB, Tomasello M. Children extend both words and non-verbal actions to novel exemplars. Developmental Science. 2003;6(2):185–190. 10.1111/1467-7687.00270

[7] Benedict H. Early lexical development: Comprehension and production. Journal of Child Language. 1979;6(2):183–200. 10.1017/S0305000900002245 468932

[8] Bergelson E, Swingley D. At 6–9 months, human infants know the meanings of many common nouns. Proceedings of the National Academy of Sciences. 2012;109(9):3253–3258. 10.1073/pnas.1113380109 PMC3295309 22331874

[9] Tincoff R, Jusczyk PW. Six-Month-Olds Comprehend Words That Refer to Parts of the Body. Infancy. 2012;17(4):432–444. 10.1111/j.1532-7078.2011.00084.x 32693484

[10] Bahrick LE, Gogate LJ, Ruiz I. Attention and Memory for Faces and Actions in Infancy: The Salience of Actions over Faces in Dynamic Events. Child Development. 2002;73(6):1629–1643. 10.1111/1467-8624.00495 12487483

[11] Hunnius S, Bekkering H. The early development of object knowledge: A study of infants’ visual anticipations during action observation. Developmental Psychology. 2010;46(2):446–454. 10.1037/a0016543 20210504

[12] Gogate L, Maganti M, Bahrick LE. Cross-cultural evidence for multimodal motherese: Asian Indian mothers’ adaptive use of synchronous words and gestures. Journal of experimental child psychology. 2014;129:110–126. 10.1016/j.jecp.2014.09.002 25285369 PMC4252564

[13] Riggs KJ, Mather E, Hyde G, Simpson A. Parallels Between Action-Object Mapping and Word-Object Mapping in Young Children. Cognitive Science. 2015;40(4):992–1006. 10.1111/cogs.12262 26110970

[14] Dysart EL, Mather E, Riggs KJ. Young children’s referent selection is guided by novelty for both words and actions. Journal of Experimental Child Psychology. 2016;146:231–237. 10.1016/j.jecp.2016.01.003 26897305

[15] Yoon EY, Heinke D, Humphreys GW. Modelling direct perceptual constraints on action selection: The Naming and Action Model (NAM). Visual Cognition. 2002;9(4-5):615–661. 10.1080/13506280143000601

[16] Chainay H, Humphreys GW. Privileged access to action for objects relative to words. Psychonomic Bulletin & Review. 2002;9(2):348–355. 10.3758/BF03196292 12120799

[17] Hahn ER, Gershkoff-Stowe L. Children and adults learn actions for objects more readily than labels. Language Learning and Development. 2010;6(4):283–308. 10.1080/15475441003635315

[18] Dysart EL. Parallels Between Word and Action Learning in 3- to 5-year-olds. University of Hull; 2018.

[19] Puccini D, Liszkowski U. 15-month-old infants fast map words but not representational gestures of multimodal labels. Frontiers in Psychology. 2012;3:101 10.3389/fpsyg.2012.00101 22493588 PMC3318184

[20] Waxman SR, Gelman SA. Early word-learning entails reference, not merely associations. Trends in Cognitive Sciences. 2009;13(6):258–263. 10.1016/j.tics.2009.03.006 19447670 PMC2829659

[21] Gogate LJ, Prince CG, Matatyaho DJ. Two-month-old infants’ sensitivity to changes in arbitrary syllable–object pairings: The role of temporal synchrony. Journal of Experimental Psychology: Human Perception and Performance. 2009;35(2):508–519. 10.1037/a0013623 19331504

[22] Robinson CW, Sloutsky VM. Auditory dominance and its change in the course of development. Child Development. 2004;75(5):1387–1401. 10.1111/j.1467-8624.2004.00747.x 15369521

[23] Fulkerson AL, Waxman SR. Words (but not Tones) facilitate object categorization: Evidence from 6- and 12-month-olds. Cognition. 2007;105(1):218–228. 10.1016/j.cognition.2006.09.005 17064677 PMC2099297

[24] Grimm H, Doil H. Elternfragebogen für die Früherkennung von Risikokindern.; 2006.

[25] Szagun G, Stumper B, Schramm SA. Fragebogen zur frühkindlichen Sprachentwicklung (FRAKIS). Frankfurt/M: Pearson. 2009.

[26] Bayley N. Bayley scales of infant and toddler development. 3rd ed San Antonio, TX: Psychological Corporation; 2006.

[27] Gogate LJ, Bahrick LE. Intersensory redundancy facilitates learning of arbitrary relations between vowel sounds and objects in seven-month-old infants. Journal of Experimental Child Psychology. 1998;69(2):133–149. 10.1006/jecp.1998.2438 9637756

[28] Matatyaho-Bullaro DJ, Gogate LJ, Mason Z, Cadavid S, Abdel-Mottaleb M. Type of object motion facilitates word mapping by preverbal infants. Journal of Experimental Child Psychology. 2014;118:27–40. 10.1016/j.jecp.2013.09.010 24211772

[29] Sloutsky VM, Napolitano AC. Is a picture worth a thousand words? Preference for auditory modality in young children. Child Development. 2003;74(3):822–833. 10.1111/1467-8624.00570 12795392

[30] Von Holzen K, Mani N. Language nonselective lexical access in bilingual toddlers. Journal of Experimental Child Psychology. 2012;113(4):569–586. 10.1016/j.jecp.2012.08.001 22980955

[31] R Core Team. R: A Language and Environment for Statistical Computing; 2016. Available from: https://www.R-project.org/.

[32] Swingley D, Pinto JP, Fernald A. Continuous processing in word recognition at 24 months. Cognition. 1999;71(2):73–108. 10.1016/S0010-0277(99)00021-9 10444905

[33] Mirman D, Dixon JA, Magnuson JS. Statistical and computational models of the visual world paradigm: Growth curves and individual differences. Journal of Memory and Language. 2008;59(4):475–494. 10.1016/j.jml.2007.11.006 19060958 PMC2593828

[34] Mirman D. Growth curve analysis and visualization using R. CRC Press; 2016.

[35] Bates D, Maechler M, Bolker B, Walker S, et al lme4: Linear mixed-effects models using Eigen and S4. R package version. 2014;1(7):1–23.

[36] Namy LL, Campbell AL, Tomasello M. The changing role of iconicity in non-verbal symbol learning: A U-shaped trajectory in the acquisition of arbitrary gestures. Journal of Cognition and Development. 2004;5(1):37–57. 10.1207/s15327647jcd0501_3

[37] Namy LL, Waxman SR. Words and Gestures: Infants’ Interpretations of Different Forms of Symbolic Reference. Child Development. 1998;69(2):295–308. 10.2307/1132165 9586206

[38] Tincoff R, Seidl A, Buckley L, Wojcik C, Cristia A. Feeling the Way to Words: Parents’ Speech and Touch Cues Highlight Word-To-World Mappings of Body Parts. Language Learning and Development. 2018; p. 1–23.30416398

[39] Junge C, Cutler A, Hagoort P. Word segmentation at ten months and word processing at 16 months. In: Neurobilingualism: Bilingual functioning from infancy to adulthood; 2009.

[40] Taxitari L, Twomey K, Westermann G, Mani N. Only cows and horses: The limits of infants’ early word learning. under review. 10.1080/15475441.2019.1670184 PMC7077354 32256251

[41] Matatyaho DJ, Gogate LJ. Type of maternal object motion during synchronous naming predicts preverbal infants’ learning of word–object relations. Infancy. 2008;13(2):172–184. 10.1080/15250000701795655 33412725

[42] Werker JF, Cohen LB, Lloyd VL, Casasola M, Stager CL. Acquisition of word–object associations by 14-month-old infants. Developmental Psychology. 1998;34(6):1289–1309. 10.1037/0012-1649.34.6.1289 9823513

[43] Roembke TC, McMurray B. Observational word learning: Beyond propose-but-verify and associative bean counting. Journal of Memory and Language. 2016;87:105–127. 10.1016/j.jml.2015.09.005 26858510 PMC4742346

[44] Waxman SR, Fu X, Arunachalam S, Leddon E, Geraghty K, Song HJ. Are Nouns Learned Before Verbs? Infants Provide Insight Into a Long-Standing Debate. Child Development Perspectives. 2013;7(3):155–159. 10.1111/cdep.12032 PMC3821773 24223064

[45] Westermann G, Mani N, editors. Early Word Learning. Routledge; 2017.

[46] Gogate L, Hollich G. Early Verb-Action and Noun-Object Mapping Across Sensory Modalities: A Neuro-Developmental View. Developmental Neuropsychology. 2016;41(5-8):293–307. 10.1080/87565641.2016.1243112 28059566

