# Supplementary material for: Word-object and action-object association learning across early development
Source: PLoS One. 2019 Aug 8;14(8):e0220317. doi: 10.1371/journal.pone.0220317 (PMC6687139; doi:10.1371/journal.pone.0220317)
Supplement: S1 Appendix — (PDF) [file pone.0220317.s001.pdf]

Word-object and action-object association learning across early development

[1,2]Sarah Eiteljörge, [3]Maurits Adam, [3]Birgit Elsner & [1,2]Nivedita Mani

[1]Psychology of Language, University of Goettingen

[2]Leibniz ScienceCampus Primate Cognition, Goettingen

[3]Developmental Psychology, University of Potsdam

Word-object and action-object association learning across early development

Appendix

Analysis of all age groups

ANOVA. For all age groups together, a 2 (Condition) x 4 (Age) ANOVA revealed a significant effect of Age (F(3,132) = 27.48, $p < .001$, $\eta_p^2 = .38$) and a tendency of Condition (F(1,132) = 2.94, $p = .089$, $\eta_p^2 = .02$), but no interaction of the two factors (F(3,132) = 1.75, $p = .161$, $\eta_p^2 = .04$).

GLMM. Comparing a model including Condition with the reduced model without Age or Condition revealed that Condition did not significantly improve the model ($\chi 2 = 4.15$, df = 5, $p = .53$). However, including Condition and Age in the interaction did improve the model significantly compared to a Condition-only model ($\chi 2 = 109.09$, df = 30, $p < .001$) or an Age-only model ($\chi 2 = 49.89$, df = 20, $p < .001$). Using drop1, the model revealed a significant interaction of Condition*Age*poly3 ($\chi 2 = 26.13$, df = 3, $p < .001$). These results suggest differences in the time course of target recognition between the two conditions across age groups.

Together, these results suggest that there are differences across age groups and differences between the two conditions, but an interaction between the two factors can only be observed when considering time within the trial as a crucial factor.
